# A population based pediatric oncology registry in Southern Sweden: the BORISS registry

**DOI:** 10.1007/s10654-018-0437-1

**Published:** 2018-09-06

**Authors:** Thomas Wiebe, Lars Hjorth, Mercedes Marotta Kelly, Helena M. Linge, Stanislaw Garwicz

**Affiliations:** 1 Faculty of Medicine, Department of Clinical Sciences Lund, Pediatrics, Lund University, Lund, Sweden; 2 Regional Cancer Center South, Lund, Sweden; 3 Department of Clinical Sciences Lund, Pediatrics, Lund University, Skane University Hospital, Lund, Sweden

**Keywords:** Childhood cancer survivorship, Registry, Population based, Sweden

## Abstract

A population based registry, with the acronym BORISS, was established. It contains all individuals (0–18 years of age at diagnosis) diagnosed with cancer from 1970–01–01 until 2016–12–31 in Southern Sweden. The treatment data has been entered into the registry after confirmation of the diagnosis by the Swedish national cancer registry and updates on vital status from the Swedish population registry. The number of individuals with a pediatric cancer diagnosed during these 46 years are 2928. Of these, 2065 are currently alive and 1882 individuals are 5-year survivors. Data on treatment and malignancy of the 5–year survivors has been collected from medical records and entered into the database. Treatment data contains surgical procedure, target organ of radiation therapy including dose and fractionation, and cytostatic treatment with dose (mg) per body surface area (m^2^) for all cytostatic agents. Data on individuals receiving stem cell treatment is included. The database is unique in that it is population based, contains all individuals and detailed treatment data on all 5-year survivors after childhood cancer in Southern Sweden since 1970. The database has contributed to several academic theses in the field of late effects after childhood cancer. BORISS also supports the Late Effect Clinic at Skåne University Hospital in Lund, Sweden with treatment details enabling a stratified surveillance.

## Introduction

Survival after childhood cancer has increased dramatically over the last 45 years. Survival past 5 years after diagnosis now averages 80% in high income countries [1]. Sweden has approximately 350 new annual cancer cases diagnosed up to 18 years of age and approximately 9000 adult long-term childhood cancer survivors (CCS). The number of CCS for Europe is estimated to be 300–500,000, and the number increases by 12,500 each year. An estimated 65% of these individuals have moderate to severe long term effects of their childhood cancer and the treatment [2]. Late effects after childhood cancer treatment is a growing field in both pediatric and adult medicine and several large research initiatives have been taken in recent years [3].

The late effects clinic at Skåne University Hospital dates back to 1987, when it became clinically apparent that cured childhood cancer patients would require long-term follow up. The clinic, a collaboration between the pediatric and adult oncology units, bases its recommendations on national guidelines and details of the primary disease. The collection of details of each individual treatment history became necessary in order to perform adequate surveillance. The collection of such information resulted in the population based BORISS registry.

## Methods

### Ethical approvals

Approval to gather data was sought and received from the central review board within the Council of Skåne. The permit also covered data gathering from patients living in the adjacent councils Blekinge, Kronoberg and Southern Halland. These three councils together with Council of Skåne constitutes Southern Sweden with a population of approximately 1.8 million inhabitants. In agreement with the laws governing quality registries, information was collected to monitor and enhance the quality of care provided at Skåne University Hospital. The law does not impose a requirement on any consent from the individual. At the time of diagnosis, the parents of the patients were informed and presented with the possibility of opting out from several quality registries.

### Data collection

All individuals in Southern Sweden with a cancer diagnosis occurring between the ages of 0–18 years of age from 1970–01–01 -2016–12–31 were identified in the national cancer registry. The starting point of enrollment of patients into the registry was chosen due to the low survival rates of childhood cancer before 1970. Since its start in 1958, Swedish law mandates that all cancer cases be reported to the national registry which registers all cancer diagnoses (primary and subsequent) but not details on treatment. The vital status and personal details of the childhood cancer patients were retrieved from the Swedish population registry. Together this data formed the basis for the manual data collection of treatment history and details of the primary malignancy found in the medical records of each individual. In order for the treatment data to be collected and entered into BORISS, the medical records were manually gathered from the archives of the children’s clinic in Lund. Records were also found in the departments of Neurosurgery, Pediatric surgery, Medicine, Oncology and Pathology in Lund and in other hospitals in Southern Sweden.

The diagnosis of a malignancy was verified by an experienced pediatric oncologist (TW), by assessment of the pathology report. The diagnosis of malignancy is coded according to ICD-7, ICD-9, C24, ICD-O version 3 and ICD-10. The registry contains 219 different histological diagnoses, divided in 12 categories according to ICCC. Details of chemotherapy were extracted from prescription documents and later on from separate chemotherapy records. The dates and administration routes of all chemotherapy was recorded. All cytostatic agents (conventional drugs and more recently introduced drugs e.g. tyrosine kinase inhibitors, protein kinase inhibitors and antibodies) were recorded. The chemotherapy drugs (n = 91) are coded by a 3-digit number. Cumulative doses of each drug (mg/m^2^ body surface area) were calculated. From the Radiation clinic, details of target organ(s), total irradiation dose (Gy) and fractionation was gathered. The target organ which received radiation therapy is coded by a 3-digit number and the target list covers 150 targets. When surgery was performed, details of the procedure, microscopic surgical margins, removal of organ(s) or an extremity, if any, were recorded. For stem cell treatment, details of conditioning therapy (with chemotherapy and/or total body irradiation), and dates of the procedure were recorded.

All entered data was cross-checked by two separate individuals. The registry contains personal data, diagnosis (in plain text and in code; ICD-7, ICD-9, C24, ICD-10 and ICD-0 version 3), and date of diagnosis. Phenotypic and cytogenetic data for leukemia, as well as cytogenetic aberrations with regards to solid tumors were gathered from medical records. Where available, data was recorded on other serious diseases, previous treatments, constitutional chromosomal aberrations, and other possible immunosuppressive treatments (pre-dating the cancer diagnosis), as was heredity for malignancies, and data on relapses. By annual updating of the database from the national cancer registry and the population registry, the development of secondary and subsequent primary neoplasms and vital status, are obtained.

Prospective collection of treatment data is carried out from 2016–01–01 and onwards under the governance of the Council of Skåne.

### Data access

Before any data can be accessed for research purposes, an approved ethical permit from the Regional Ethical review board, as well as a permission from the Council of Skåne must be obtained. Data can be made available through collaboration with researchers affiliated with Skåne University Hospital or Lund university based on their knowledge of the data, its collection, purpose, and limitations. The cross-border sharing of personal data is under review due to the General Data Protection Regulation all inquiries must take into account the developments in interpretation of the new regulation. Treatment details are available to the late effects clinic in Lund as part of regular follow-up health care.

### Technical aspects

The database was originally built as a Microsoft Access database with 2 parts: an application for data entry and a separate database for data storage. Controls of data entries were put in place for quality assurance of individual fields and to maintain coherence between different fields. The database storage was placed on a central server with restricted access. The database was modernized to an SQL database in 2017, and a new web based registration form was constructed preserving the original structure. An activity log was added to keep track of registration activities. The data is secured based on user membership in active directory user groups.

## Results

The population-based BORISS registry contains all individuals with a first primary neoplasm diagnosed between 1970–01–01 and 2016–12–31 being 0–18 years of age at diagnosis, in total 2928 individuals. It contains complete treatment data on 2065 currently living individuals out of which 1882 individuals who are 5-year survivors. The oldest living person whose data is registered was born in 1953. The distribution of diagnoses according to ICCC [4], among the 5-year survivors (n = 1882) and the gender distribution is shown in Table 1. Descriptive data of the distribution of the survivors’ ages (0–65 years of age) and time since diagnosis (5–47 years) is shown in Table 2. Table 2 also shows the number of individuals which have received different treatment modalities.

**Table 1:** Number of 5-year survivors according to diagnostic group

Diagnostic group	ICD-10	5 year survivors (n =)	
		Total	Female
		1882	855
*Group I Leukaemias*			
Ia Acute lymphoid	C91.0, C91.7	329	144
Pre-B		243	111
B cell		8	0
T-cell		30	5
Non-specified		48	28
Ib Acute myeloid	C92.0, C92.3, C92.4, C92.5, C92.9, C93.0, C93.7, C94.0, C94.2	55	32
Ic Chronic myeloid	C92.1	5	5
*Group II Lymphomas*			
IIa Hodgkin lymphomas	C81.0–C81.9	110	46
IIb Non-Hodgkin lymphomas	C83.0–C83.9, C84.5, C85.8, C86.6 except C83.7 (Burkitt)	43	14
IIc Burkitt lymphomas	C83.7	35	7
IId Lymphoreticular neoplasms. Langerhans cellhistiocytos	C96.0	42	17
*Group III Central nervous system*		492	213
Intracranial	C71.0–C71.9	272	125
Intraspinal	C72.0–C72.9	64	22
Meningeas	C70.0–C70.1	27	12
Opticus gliomas	C72.3	33	8
Acusticus gliomas	C72.4	8	5
Hypophyseal adenomas	C75.1	35	23
Hypophyseal craniopharyngeomas	C75.2	34	13
Corpus pineal neoplasms	C75.3	19	5
*Group IV Neuroblastoma*		76	31
Neuroblastoma in sympathetic nervous ganglion	C47.0–C47.9	54	24
Neuroblastom in adrenal medulla	C74.1	22	7
Sarcomas of peripheral nerves	C47.0–C47.9	21	11
*Group V Retinoblastoma*	C69.2	41	21
*Group VI* Renal tumours	C64.9	92	51
Wilms’ tumour	C64.9	83	46
Other renal tumours	C64.9	9	5
*Group VII* Hepatic tumours	C22.2	11	6
Hepatoblastoma	C22.2	11	6
*Group VIII* Malignant bone tumours		94	44
VIIIa Osteosarcomas	C40.0–C40.9 C41.0–C41.9	34	17
VIIIb Chondrosarcomas	C40.0–C40.9 C41.0–C41.9	3	1
VIIIc Ewing sarcomas (bone and soft tissue)	C40.0–C40.9 C41.0–C41.9 C49.0–C49.9	33	17
VIIId Other malignant bone tumours	C40.0–C40.9 C41.0–C41.9	24	9
*Group IX Soft tissue sarcomas*	C49.0–C49.9	147	58
Rhabdomyosarcomas	Embryonal	27	9
	Alveolar	7	4
	Non-specified	2	2
Other soft tissue sarcomas		111	43
*Group X Germ cell tumours*		73	33
Xa Intracranial and intraspinal germ cell tumours		Included in group III	
Xb Germ cell tumours (not intracranial, intraspinal, ovary nor testis)	C49.0–C49.9	24	15
Xc Malignant germ cell tumours			
In testis	C62.0–C62.9	31	0
In ovary	C56.9	18	18
*Group XI Other malignant epithelial neoplasms and malignant melanomas*		103	68
XIa Adrenal cortical carcinoma	C74.0	3	1
XIb Thyroid carcinomas	C73.9	52	41
XIc Nasopharyngeal carcinomas	C11.9	4	1
XId Malignant melanomas	C43.0–C43.9	44	25
XI e–f		Included in Group XII	
*Group XII Other malignant neoplasms*		96	54

**Table 2:** Distribution of all survivors according to age groups, time since diagnosis, and number of individuals according to treatment modality

Distributions all survivors	Total (female)	
	2 065 (968)	
	*Age Groups*	*(total n =)*
	0–14	334
	15–19	248
	20–40	960
	40–65	523
	*Years since diagnosis*	*(total n =)*
	5–9	338
	10–14	292
	15–19	253
	20–24	238
	25–29	206
	30–34	196
	35–39	198
	40–44	102
	45–47	59
	*5–47* *years since diagnosis Sum:*	*1 882*
	*Not yet 5-year survivors. Sum:*	*183*
*Treatment modality*	*Total number (female)*	
Surgery (including biopsies)	1441 (675)	
Radiotherapy	594 (263)	
Chemotherapy	1049 (475)	
Stem cell transplantation	94 (42)	
Autologous	39 (16)	
Allogenic	55 (25)	
Monoclonal Antibodies (*Mabthera, Avastin, Cetuximab, Gemtuzumab)*	12 (5)	
Tyrosine kinase inhibitors *(Erlotinib, Imatinib, Dasatinib, Nilotinib)*	11 (6)	

## Discussion

Southern Sweden has had an active presence in pediatric oncology since the late 1960s. Retrospective collection of data from childhood cancer patients for this study was time consuming, especially for the early decades where procurement of the paper charts was necessary. A single chart could take up to 8 h to extract data from. The chemotherapy records remained as paper documents until recently when digitalization became a step forward. The work was facilitated by well-organized staff at the regional archives, where the medical charts are stored.

The BORISS registry has already contributed to several academic theses at Lund University, Sweden [5–8]. BORISS also supports the late effects clinic at Skåne University Hospital in Lund, Sweden with treatment details enabling a stratified surveillance. In comparison to other cohorts, BORISS covers a region (rather than a country) with the advantage of being population-based building on the by-law-mandated national cancer registry (all cancers).

On a national level, a registry with a focus on treatment data and long-term follow-up was started in 2012 but to date it contains only 1/3 of eligible patients with coverage starting only in the 1980s. At the international level several initiatives aim at providing childhood cancer survivors with adequate and appropriate long-term medical follow-up. The EU-funded project PanCareSurFup (2011–2017) established a large cohort (n = 115,000) of childhood cancer survivors with almost 84,000 five-year survivors [9]. On a subset of these, treatment data was collected from historic medical charts with the focus of examining cardiotoxicity, late mortality and second primary neoplasms. The individuals were identified by outcome and then traced back to historical records.

In Scandinavia, a large project, Adult Life after Childhood Cancer in Scandinavia (ALiCCS) is also aimed at examining late effects in childhood cancer survivors [10]. The focus areas include diabetes mellitus, cardiopulmonary disorders, endocrine disorders and renal and gastrointestinal disorders, and as in the other recent initiatives the treatment data has had to be collected retrospectively on a patient-by-patient basis. The data collection phase (2010–2015) is finished and analyses are ongoing. The outcome groups will be compared to a statistically selected sub-cohort representing the entire population of CCS in the Nordic countries.

In the US, there are several cohorts of long term survivors after childhood cancer [2, 11]. These cohorts have contributed with valuable knowledge to the late effects field but the findings may be limited in impact as they are not population based. They can hence only suggest the true number of individuals to be expected to be affected by late effects.

### Limitations and future prospects

The number of individuals with rare cancer diagnoses in BORISS will, as expected, be small. Specific consideration should be given when conducting studies, in order not to over-interpret any findings. Other potential limitations of the data in BORISS include the quality of the information in the medical records, in particular family medical history. Complications of treatments e.g. graft-versus-host disease, surgical complications, number of placements of central administration route devices, and details of blood transfusions are not included. Non-typical childhood cancers like malignant melanoma and thyroid cancers were included in the database whereas cervical dysplasias and *mola hydatiformis* were excluded.

The BORISS registry was formed as a quality registry and this, together with data retrieval from the by-law mandated entry into the national cancer registry, is the explanation for the complete coverage rate. The longitudinal BORISS cohort will enable further research within various fields in pediatric and adult medicine. Ongoing studies aim to determine details of cardiomyopathy and cognitive effects after childhood cancer treatment. By use of artificial intelligence, an ongoing study aims to determine previously unknown associations between treatment history and outcomes.

### Concluding remarks

Motivated by the clinical need to readily be able to access the primary treatment data, and the vision of facilitating research of the underlying causes of late effects, the collection of data resulted in a population based registry in Southern Sweden. Key factors in establishing the registry were: the use of personal identification numbers in Sweden, the by law mandated entry of cancer diagnoses into the national cancer registry and the foresight of the pioneers in the field with a long-term dedication to finalize the task.
